# Supplementary material for: Assessing Preparedness and Preventive Measures for Managing Food Allergy and Anaphylaxis in Primary Schools of Rabigh, Saudi Arabia
Source: Int J Environ Res Public Health. 2025 Aug 29;22(9):1357. doi: 10.3390/ijerph22091357 (PMC12469751; doi:10.3390/ijerph22091357)
Supplement: Supplementary file 1 [file ijerph-22-01357-s001.zip › ijerph-3787760-supplementary.pdf]

Supplementary materials

Table S1 compares preparedness measures for allergic reactions between trained and untrained schools. All trained schools (100%) and most untrained schools (90.9%) reported having a communication system in place for emergencies (*p*=0.579). The assignment of a designated staff member for allergic cases was reported equally by both groups (87.5%, p = 0.338). The identification of staff roles during allergy emergencies was reported more frequently by trained schools (62.5%) compared to untrained schools (36.4%), although this difference was not significant (p = 0.790). Interestingly, a higher proportion of untrained schools (72%) than trained schools (50%) reported awareness of Pupils with food allergies or anaphylaxis (*p*=0.377). Preparedness for allergic reactions in children without a prior history of allergies was similarly low in both groups (*p = 1.000*).

Table S1 Comparison of Preparedness Measures for Allergic Reactions: Trained vs. Untrained Schools

Questions	Trained Participants (n=8)	Untrained Participants (n=11)	Total	P-value
Developing communication systems within the school that are simple to follow in emergencies	8(100.0%)	10(90.9%)	18(94.7%)	0.579[b]
Assign one staff member to deal with allergic cases?	7(87.5%)	7(87.5%)	14(73.7%)	0.338[b]
Identifying the role of each school staff member in an allergy emergency?	5(62.5%)	4(36.4%)	9(47.4%)	0.790[b]
Knowing the Pupils with food allergies or anaphylaxis	4(50.0%)	8(72.0%)	12(63.2%)	0.377[b]
Preparing for allergic reactions in children without a previous history of allergies?	3(37.5%)	4(36.4%)	7(36.8%)	1.000[b]

[a] Chi-square and [b] Fisher's Exact Test were used. Data are displayed as numbers (%). The value listed in bold indicates statistical significance (**P-value<0.05).*

Table S2 compares the implementation of preventive measures for allergic reactions between trained and untrained schools. Although trained schools reported higher adherence to most preventive measures, none of the differences reached statistical significance. Guidance for staff handling food allergies was reported by 75% of trained schools and 45.5% of untrained schools (*p = 0.428*). Close supervision of high-risk Pupils during mealtimes was noted in 25% of trained schools and 36.4% of untrained schools (*p = 0.683*). A policy against food sharing was more common among trained schools (50%) than among untrained schools (18.2%), although this difference was not statistically significant (p = 0.507). A no-nut policy was reported in 75% of trained schools and 63.6% of untrained schools (p = 1.000). Special supervision for high-risk Pupils on school buses was low in both groups, with no significant difference (p = 0.843).

Table S2 Comparison of Preventive Measures for Allergic Reactions: Trained vs. Untrained Schools

**Questions**	Trained Participants (n=8)	Untrained Participants (n=11)	**Total**	**P-value**
Is there guidance available for staff on preventing food allergies when handling food?	6(75.0%)	5(45.5%)	11(57.9%)	0.428[b]
Are high-risk Pupils being supervised more closely during mealtimes?	2(25.0%)	4(36.4%)	6(31.6%)	0.683 [b]
Is there a policy against food sharing among Pupils at your school?	4(50.0%)	2(18.2%)	6(31.6%)	0.507 [b]
Do Pupils at your school have a no-nut policy?	6(75.0%)	7(63.6%)	13(68.4%)	1.000 [b]
Is there special supervision for high-risk Pupils on school buses?	2(25.0%)	2(18.2%)	4(21.1%)	0.843 [b]

[a] Chi-square and [b] Fisher's Exact Test were used. Data are displayed as numbers (%). The value listed in bold indicates statistical significance (**P-value<0.05).*
